# Practical Implications of Myocardial Viability Studies

**DOI:** 10.5935/abc.20180051

**Published:** 2018-03

**Authors:** Wilter dos Santos Ker, Thais Helena Peixoto Nunes, Marcelo Souto Nacif, Claudio Tinoco Mesquita

**Affiliations:** 1 Setor de Medicina Nuclear - Hospital Universitário Antônio Pedro (HUAP) - Universidade Federal Fluminense (UFF), Niterói, RJ - Brazil1; 2 Serviço de Radiologia - Hospital Universitário Antônio Pedro (HUAP) - Universidade Federal Fluminense (UFF), Niterói, RJ - Brazil2

**Keywords:** Tissue Survival, Diagnostic Imaging, Myocardial Revascularization / surgery, Myocardium Stunning / physiopathology

## Abstract

Many non-invasive methods, such as imaging tests, have been developed aiming to add a contribution to existing studies in estimating patients’ prognosis after myocardial injury. This prognosis is proportional to myocardial viability, which is evaluated in coronary artery disease and left ventricular dysfunction patients only.

While myocardial viability represents the likelihood of a dysfunctional muscle (resulting from decreased oxygen supply for coronary artery obstruction), hibernation represents post-interventional functional recovery itself.

This article proposes a review of pathophysiological basis of viability, diagnostic methods, prognosis and future perspectives of myocardial viability. An electronic bibliographic search for articles was performed in PubMed, Lilacs, Cochrane and Scielo databases, according to pre-established criteria.

The studies showed the ability of many imaging techniques in detecting viable tissues in dysfunctional areas of left ventricle resulting from coronary artery injuries. These techniques can identify patients who may benefit from myocardial revascularization and indicate the most appropriate treatment.

## Introduction

Assessment of myocardial viability using non-invasive imaging techniques has motivated several studies in search of the most promising and sensitive tests. These tests highlight the importance of a correct evaluation of this condition for an appropriate risk stratification and selection of patients considered eligible for myocardial revascularization. Since cardiac function is not a dichotomous variable, some of its aspects measured by imaging techniques may not be measurable by another method. Useful parameters to guide therapeutic strategies include ejection fraction, scar size, ischemia and remodeling extension, as well as duration of cardiac dysfunction.^1,2^

Using a multimodal approach of viability, a pilot study^3^ showed higher values for these variables, which were analyzed in combination, providing a more reliable characterization of myocardial function. However, due to the lack of larger studies, imaging tests based on multimodal approach are not recommended yet. It is worth pointing out that even though the presence of a viable myocardium in a large heart area is important for revascularization, the decision for this procedure should be based on patient’s clinical status, evidence of ischemia, coronary anatomy and left ventricular global and regional function.^4^

Determination of myocardial viability is a common and clinically relevant challenge, that may be necessary in post-infarction patients receiving thrombolytic therapy. Also, it may be helpful for surgeons and cardiologists in choosing the best therapy from interventionist strategy, angioplasty and myocardial revascularization.^5^ This is particularly important in cases when myocardial revascularization is considered, due to high mortality rate and perioperative morbidity in these patients.^6^

In viability studies, while nuclear medicine techniques have high sensitivity, the techniques used to evaluate contractile reserve have higher specificity. Imaging methods, such as computed tomography (CT), positron-emission tomography (PET), myocardial scintigraphy, echocardiography with dobutamine and cardiac magnetic resonance (CMR) have been exhaustively investigated in attempt to establish the best method for myocardial study.^7^

### Pathophysiology

**Figure 1:**
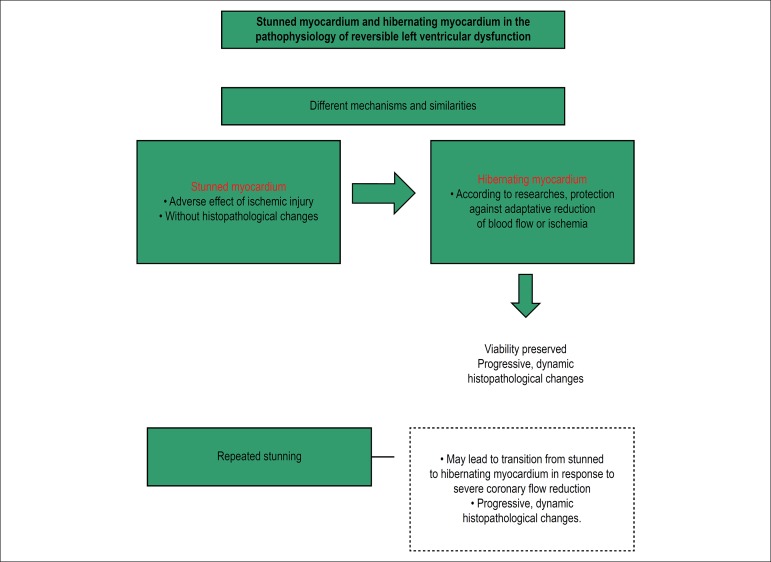
Main feature of the physiopathology of stunned myocardium and hibernating myocardium [adapted from Chareonthaitawee et al.^8^]

Myocardial viability refers to myocardial cells that are alive after myocardial injury, according to cellular, metabolic and contractile functions. It describes ventricular dysfunction without tissue necrosis, which enables functional recovery after restoration of blood supply. In this context, although the definitions “stunned myocardium” and “hibernating” myocardium have distinct characteristics, the latter may represent the adaptation of repeated episodes of the former, as described by Chareonthaitawee et al.^8^ (Figure 1).

“Stunned myocardium” results from a rapid, severe episode of coronary occlusion followed by recovery of coronary flow. An abrupt decrease in coronary flow causes contractile dysfunction, which persists even after its restoration. Despite minimal necrosis, ventricular dysfunction may be prolonged, from hours or even weeks. A group of researchers,^9^ investigating ventricular function in patients with coronary heart disease, demonstrated that repeated episodes of ischemia may lead to cumulative stunning, which contributes to the development of chronic, post-ischemic, left ventricular dysfunction. Interestingly, similar degrees of left ventricular dysfunction in distinct patients may be associated with significant differences in the degree of myocardial viability. Besides, viability is not correlated with myocardial wall thickness, since ventricular wall thinning does not necessarily mean absence of myocardial viability.^10^

“Hibernating myocardium” has been defined as the presence of severe systolic dysfunction with evidence of hypoperfusion at rest;^3^ it refers to a myocardium with preserved cellularity, but reduced blood flow, leading to depressed ventricular function, even at rest.^11^ The first theory of hibernating myocardium characterized it as an adaptation to chronic hypoperfusion whose intensity was not sufficiently significant to cause infarction.^12^ This was supported by CMR and PET studies on dysfunctional myocardial areas with reduced blood flow.^13,14^ However, pathogenesis of hibernating myocardium is still subject of studies and has not been elucidated yet,; it is believed, however, to be conditioned to a functional dysregulation related to mitochondrial impairment, in attempt to protect cardiac muscle cells from ischemia.^11,15^ Hibernating is also known to have intrinsic cellular and extracellular changes, that may be associated with the time required for reversibility of the process,^12^ which may vary from days to 14 months.^16,17^

### Clinical implications

Assessment of viability may significantly contribute to the identification of patients who would benefit from revascularization, particularly by the improvement in ventricular function and survival. To demonstrate the clinical usefulness of viability, a meta-analysis was performed with 24 studies on different techniques on viability detection in patients with chronic coronary artery disease (CAD) and myocardial dysfunction. Annual mortality rate in the group of patients with myocardial viability and in drug treatment was 16%, in contrast with 3.2% in the group that underwent revascularization.^18,19^

In CAD patients, left ventricular dysfunction may be caused by areas of viable myocardium and fibrotic areas combined. Assessment of cardiac muscle using imaging methods enables the localization, quantification of viability in dysfunctional myocardium and possibility of anatomical revascularization, which is essential for treatment planning of these patients.^11^

This article proposes a review of pathophysiological bases of myocardial viability, diagnostic methods available, prognosis and risk for this condition. A bibliographic search was performed on the electronic databases PubMed, Lilacs, Cochrane and Scielo, based on pre-established criteria.

## Methods

To achieve the objectives and results proposed, a descriptive review of scientific literature was conducted of studies on diagnostic accuracy of imaging tests used for the measurement of myocardial viability. We included both studies showing the superiority of certain method and those comparing the efficacy of the methods from the perspective of other authors.

### Inclusion criteria:

Types of study: as “review articles”, we included studies aimed to demonstrate the efficacy of imaging tests in measuring myocardial viability after ischemia.

Population: heart disease patients with history of myocardial infarction.

### Exclusion criteria:

Studies that did not provide a detailed description of the protocols of the diagnostic methods or of data statistical analysis, and studies that did not meet the inclusion criteria were excluded.

### Search on the databases

#### The following databases were searched:

- PubMed/MEDLINE: North-American database, one of the largest in health, with no limits of date.

The following descriptors were used for the search on Pubmed: Myocardial viability; PET; CT; SPECT; Resonance Magnetic myocardial; Echocardiography.

- LILACS: database that integrates the BIREME system and includes several scientific journals, dissertations and books.

The following terms were used: Myocardial Viability ANS viability studies.

- COCHRANE: database focused on systemic reviews. The terms used in this database were: Myocardial viability.

### Diagnostic methods

#### Assessment of myocardial viability by dobutamine stress echocardiography

The use of dobutamine stress echocardiography for detection of myocardial viability is an efficient and safe method in both acute and chronic phases of CAD,^20^ with low incidence of significant events^21^ (around 0,5%).^21,22^ This method has favorable sensitivity (77-89%) and specificity (68-93%) not only in the post-infarction phase,^23,24^ but also in the chronic phase (82% and 92%, respectively), as shown by Marzullo et al.^25^

#### Assessment of myocardial viability using baseline-nitrate ^99m^Tc-Sestamibi scintigraphy

Myocardial perfusion scintigraphy using nitrate-augmented ^99m^Tc-Sestamibi is a widely available method for assessment of myocardial viability. The use of nitrates enables the improvement of blood flow in narrowed and collateral vessels, responsible for irrigation of hypoperfused areas, which potentiates the ability of the method to detect viable tissues, especially when combined with ^99m^Tc-Sestamibi.^11^ This is assured by the fact that both absorption and retention of sestamibi depend on perfusion, cell membrane integrity and membrane potential (mitochondrial function), which hence constitute the markers of viable tissue.^25-27^ Schinkel AF et al.^28^ reported a 81% sensitivity and 69% specificity of nitrate-enhanced ^99m^Tc-Sestamibi scintigraphy to detect viability, which is lower than those reported with the use of PET-^18^F-FDG.^28^ In Figure 1, we illustrate a case where rest perfusion defect, initially attributed to the infarction area, normalized after treatment of the coronary obstruction in the anterior descending artery, demonstrating a viable myocardium. These findings illustrate a practical limitation of imaging techniques using ^99m^Tc-Sestamibi in the detection of myocardial fibrosis and viability.

**Figure 2:**
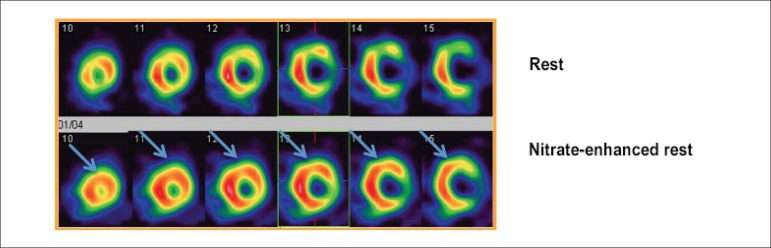
Images of rest (upper line) and nitrate-enhanced rest (lower line) myocardial perfusion scintigraphy, showing improvement of perfusion in anterior (apical, medial and basal) and anterolateral (medial and basal) segments.

In most studies on baseline-nitrate ^99m^Tc-Sestamibi scintigraphy, two patterns of images are commonly obtained: rest images and nitrate-enhanced images. Reversibility of the lesion (by filling) is indicative of viability. Sciagra et al.^29^ studied 105 patients with chronic CAD and left ventricular dysfunction who underwent baseline-nitrate sestamibi perfusion imaging and showed that the most powerful prognostic predictors of events were the number of nonrevascularized dysfunctional areas with viability in sestamibi imaging^28,29^ (Figure 2).

#### Assessment of myocardial viability with ^201^Tálio

^201^Tálio has some limitations for routine use, due to its longer physical half-life, and relatively low photon energy and flow. This may yield images with low count-rates and possible attenuation artifacts and, consequently, suboptimal images.^4^

**Figure 3:**
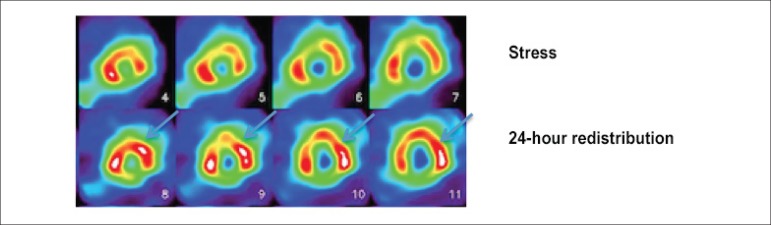
Myocardial perfusion scintigraphy with ^201^Tálio for assessment of myocardial viability; stress imaging (upper line) and 24-hour redistribution imaging after injection of the radiotracer ^201^Tálio (lower line), showing improvement of perfusion in anterior (apical, medial and basal) and anterolateral (medial and basal) segments.

^201^Tálio perfusion scintigraphy may show different perfusion defects that vary within a range from totally reversible to irreversible, according to the degree of improvement in the activity of late images.^7^

However, ^201^Tálio has the advantage of entering myocardial cells by active transportation, which increases its accuracy for detecting viable myocardium. For this purpose, two protocols are usually used - stress-redistribution-reinjection and rest-redistribution imaging. While the first is focused on data about stress-induced ischemia and viability, the second focuses only on viability^26^ (Figure 3).

In a meta-analysis, Schinkel et al. reported an 87% sensitivity and 54% specificity in predicting post-revascularization recovery.^28^ Some studies have suggested that improvement in systolic function is not a *sine qua non* for clinical benefits, with a better prognosis but no improvement in the ejection fraction of some patients.^4,11,26,28^

### Assessment of myocardial viability using positron emission tomography with fluorine-18-deoxyglucose (^18^F-FDG PET)

Among the methods available for assessment of myocardial viability, ^18^F-FDG PET is considered the gold standard method.^30,31^ Because ^18^F-FDG is a glucose analog, it is used to evaluate the metabolism of cardiac glucose, and thereby the uptake of this marker is similar to glucose utilization by myocytes.^4^

In fasting conditions, myocardium uses preferentially free fatty acids as energy source, whereas in post-prandial phase, its metabolism is shifted to glucose (with increased levels of circulating insulin).^5^ As the metabolism of free fatty acids depends on oxygen, during myocardial ischemia, glucose is the preferred substrate (glycolytic pathway), which is the hallmark of myocardial viability.^35,32-35^

**Figure 4:**
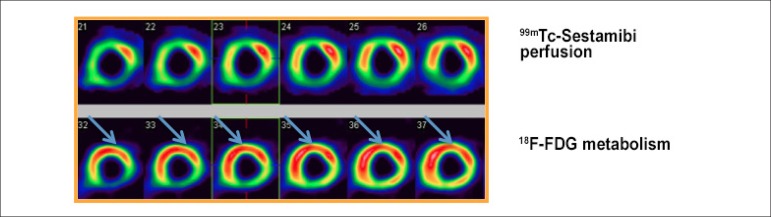
Myocardial perfusion scintigraphy with ^99m^Tc-Sestamibi (upper line) and ^18^F-FDG PET (lower line) for assessment of myocardial viability, showing improvement in perfusion/metabolism in anterior (apical, medial and basal), apical septal, anteroseptal (medial and basal) and inferoseptal (medial and basal) segments; “mismatch” pattern.

PET with ^18^F-FDG has mean sensitivity of 92% and specificity of 63% in assessing the likelihood of functional improvement of the muscle in the after revascularization. Many studies have used comparative data of perfusion and ^18^F-FDG uptake, defining myocardial viability as hypoperfused areas with preserved glucose metabolism.^26,28,32-34^ (Figure 4).

Overall improvement of left ventricle may also be evaluated by ^18^F-FDG. Left ventricular ejection fraction (LVEF) improves from 37% to 47% (mean values) in patients with myocardial viability detected by ^18^F-FDG PET after revascularization. In patients without viable myocardium, LVEF remained almost unchanged (39% x 40%).^31,34-39^

### Assessment of myocardial viability with computed tomography (CT)

CT is the most recent and widely used method for coronary angiography. Three techniques are currently used for cardiac CT - coronary angiography, CT with iodinated and non-contrast CT - and all of them can provide information on myocardial viability.^40-42^

CT coronary angiography has high negative predictive value (> 95%) in excluding epicardial CAD, with increasing role in the assessment of chest pain. It may also provide valuable information in the evaluation of patients with left ventricular systolic dysfunction, with suspected congenital heart disease or coronary anomaly.^42^

Delayed enhancement CT uses a similar principle to gadolinium-based magnetic resonance (MR) imaging for imaging studies of myocardial scarring. In CT, the use of iodinated contrast causes an increase in Hounsfield units in contrasted tissues, due to attenuation of X-rays, allowing the visualization of cardiac muscle in the early arterial phase, and discrimination of macro and microvascular obstruction. When evaluated 5-10 minutes after injection of iodinated contrast and increased enhancement, the obstruction is suggestive of infarction, due to extracellular contrast accumulation.^41,42^

Finally, non-contrast CT can reveal calcified aneurysms in the left ventricle, for showing similar images to those obtained during attenuation correction scans or calcium scoring.^41,42^

Some advantages of cardiac CT include the possibility of being performed in combination with coronary CT, requiring only the addition of some minutes to the angiography protocol; its high spatial resolution, being of great importance in evaluation of small infarctions; near-isotropic resolution and reliable 3D data reconstruction for the small slide thickness; possibility of inclusion of patients with pacemakers and other metallic devices. As disadvantages, we can mention the necessity of higher radioactive emission for acquisition of additional images following coronary images, and poorer localization ability and transmurality as compared with CMR.^43^

### Assessment of myocardial viability with MR imaging

MR is a highly efficient method for myocardial viability study^26^ and has played an important role in the clinical practice. It has also been considered a gold standard method in the assessment of left ventricular function. MR allows assessment of left ventricular dysfunction associated with chronic ischemic disease by evaluation of contractile reserve using dobutamine at low dose and, most importantly, evaluation of fibrosis by late gadolinium enhancement. In a metanalysis, Romero et al.^44^ concluded that MR with low-dose dobutamine has high sensitivity and specificity (81% and 91%, respectively), whereas late gadolinium enhancement MR has 95% sensitivity and 51% specificity, and high accuracy in determining some parameters, including ejection fraction, left ventricular volume, regional wall motion, and myocardial thickness.^45,46^ Left ventricular wall thickness at end diastole is important to exclude viability.

The most notable characteristic of MR is its high spatial resolution, and, for this reason, the method stands out for its high imaging quality and capacity to diagnose ischemic areas that would not be detectable by other methods. MR may also be particularly useful in the assessment of myocardial blood flow at rest in hibernating areas of narrowed coronary artery and improvement of local myocardial contractility after coronary revascularization.^13,47^

**Figure 5:**
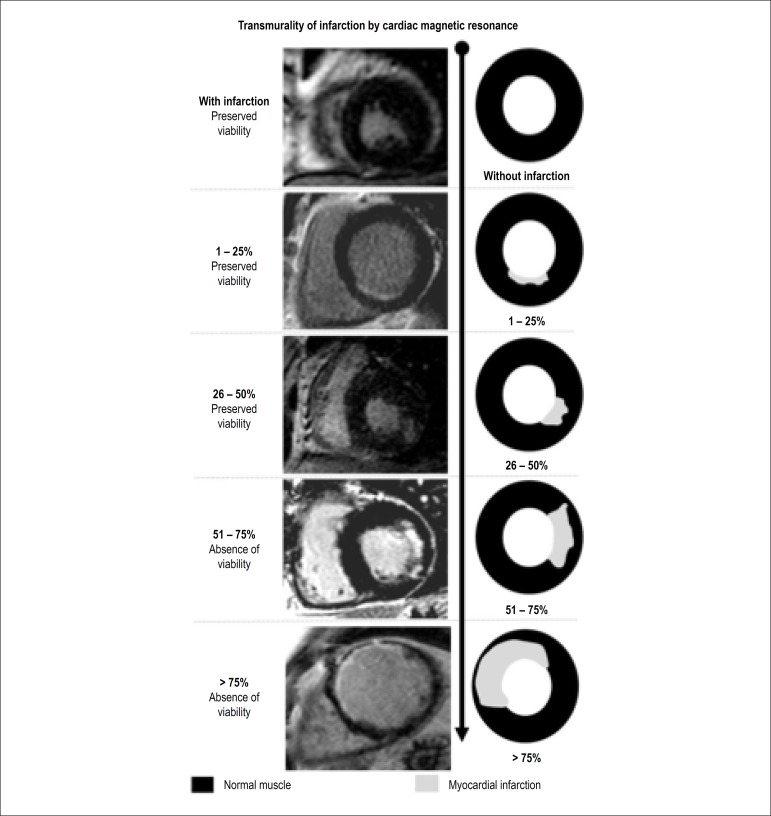
Patterns of transmurality of infarction in the presence and absence of viability by cardiac magnetic resonance

The use of gadolinium as a contrast medium in MR allows the detection of the effects of perfusion, microvascular obstruction and differentiation between transmural and subendocardial necrosis.^48^ Gadolinium has a low risk of nephrotoxicity, except for patients with end-stage renal disease, in which the risk of systemic toxicity is real. Although chelated-gadolinium compounds are distributed in the extracellular space, and do not penetrate in intact cells, they may accumulate in myocytes with ruptured cell membrane (e.g. acute myocardial infarction) and fibrotic areas^10^ (Figure 5).

The likelihood of functional recovery after revascularization is proportional to the transmurality of acute myocardial infarction. A very important marker of improvement of myocardial function is the amount of delayed enhancement by MR imaging since there is a progressive improvement in myocardial function with the increase of transmurality of scar tissue. Kim et al.^46^ evaluated the ability of contrast-enhanced MR imaging to predict functional recovery after revascularization. Approximately 80% of segments with less than 25% of transmural fibrosis had functional recovery after revascularization, whereas only 10% of the segments with transmurality higher than 50% recovered after revascularization. Selvanayagam et al.^47^ showed that delayed-enhancement cardiovascular MR imaging is a strong predictor of myocardial viability after surgical revascularization.

Left ventricular wall thickness may reveal valuable information about viability. Schinkel et al.^26^ showed that segments with an end-diastolic wall thickness of less than 5 mm was associated with higher likelihood of recovery after revascularization.

Taken together, these findings suggest that segments with an end-diastolic wall thickness of less than 5.5 mm never show recovery of function after revascularization, which may be related to the presence of nontransmural infarction. These segments contain subendocardial scar tissue, with residual viability in the epicardium. Therefore, significant wall thinning indicates scar tissue, with low likelihood of recovery after revascularization; nevertheless, evidence suggests that recovery of function may occur, but only when contrast-enhanced MR excludes scar tissue.^10^

Geber et al.^49^ demonstrated that cardiac MR was important in identifying patients with ischemic cardiomyopathy and severe left ventricular dysfunction who would benefit from myocardial revascularization. CMR can be performed in ischemic cardiomyopathy with left ventricular dysfunction to characterize myocardial viability.^50^ Limitations of this technique, however, include its high cost, difficulty of performing scans in patients with implanted devices, and limited availability.^10^

## PET-RM

A new technique - PET-MR started to be studied, but still has limited availability. The method has the advantage of combining the high spatial resolution of MR with the sensitivity of PET, without excessive ionizing radiation. In contrast to PET-CT, however, the synergism between PET and MR still need to be evaluated.

Comparison of left ventricular end-diastolic wall thickness on MRI with glucose use on ^18^F-FDG PET demonstrated that regions with an end-diastolic wall thickness of less than 5.5 mm had reduced glucose use, whereas regions with a wall thickness of 5.5 mm did not use this carbohydrate.^51^ Studies on usefulness of PET-MR in cardiology are still ongoing, but it includes specific localization of lesions, contributing to therapeutic intervention.^52^ Preliminary data indicate the possibility of PET-MR to measure inflammatory response to myocardial infarction and neoangiogenesis.^52,53^ While MR is helpful in the analysis of scar extension, PET provides characteristics of the subepicardium and likelihood of functional recovery of areas free of scars.^51^

### Comparison between the techniques:

For practical purposes, the most appropriate methods for viability assessment are those in which the clinician or the institution have the highest experience. Echocardiography with dobutamine has, in general, high positive predictive value, and thus, is relatively more specific whereas nuclear medicine techniques are more sensitive to diagnosis, with a significative negative predictive value, as can be seen in the study by Panza et al.,^54^ who compared the echocardiography and ^201^Tálio myocardial scintigraphy methods. Hakimeh et al.^55^ evaluated viable kinetic segments by resting ^(99m)^Tc-Sestamibi, and observed that the number of these segments was significantly greater than those showing a contractile response to dobutamine. Hence, due to its greater accessibility, echocardiography may be the method of choice in the screening for the presence of viability, and in a second line of investigation, a nuclear method could be used.^56^

^(99m)^Tc-Sestamibi has been used as an alternative to ^201^Tálio for its higher quality combined with lower exposure to radiation. In cases when ^(99m)^Tc-Sestamibi imaging are not conclusive, or when greater viability is still clinically possible, the use of ^201^Tálio is indicated for its higher detection rate, especially in severe hypoperfusion areas.^25^

An excellent method for assessment of hibernating myocardium is ^18^F-FDG PET, for its higher sensitivity in detecting dysfunctional, but viable, myocardium. Although a sensitivity of 93% was shown for this technique in a metanalysis,^35^ other authors reported a lower specificity (58%).^57-59^

With respect to MR and nuclear medicine techniques, comparison of contrast MR imaging, with dobutamine echocardiography and ^201^Tálio rest-redistribution showed an agreement of 83% and 75%, respectively.^60^ Klein et al.^51^ showed a good agreement between contrast MR and ^18^F-FDG PET; in patients with CAD and severely reduced LVEF, MR imaging can identify fibrotic areas with results similar to those obtained by PET measurements, provided by comparison of flow and glucose metabolism. MR also provides other parameters of tissue viability, such as wall thickness, contractile reserve and delayed enhancement.^59-62^

In addition, in comparison with CT, MR has higher contrast resolution for soft tissues, without requiring radiation exposure. CT and PET^41^ may be an alternative test to MR for patients with pacemakers, implantable cardioverter defibrillator or mechanical cardiac valve. Table 1 summarizes the comparison between these methods of assessment of myocardial viability.

**Table 1:** Comparison between myocardial viability assessment methods

	Radiation dose	Contrast/tracer redistribution	Protocol duration	Contrast phases *	Sensitivity	Specificity
Dobutamine echocardiography	n/a	n/a	30 min	n/a	77-89%	68-93%
^99m^Tc-Sestamibi SPECT	Moderate	Absent	90 a 120 min	Two injections	81%	69%
^201^Tálio SPECT	High	Present	3h with additional 24h imaging if necessary	One injection	87%	54%
^18^F-FDG PET	Moderate	Absent	1h	One injection	92%	
Delayed enhancement /coronary computed tomography angiography	Moderate	Absent	25 minutes	Two injections	n/a	n/a
Cardiac magnetic resonance	n/a	Absent	35 minutes	Two injections	92-95%	51-89%

n/a: non-applicable; DS: dobutamine-induced stress; Gad: gadolinium delayed enhancement.

* Contrast phases are correlated with better evaluation when the contrast is injected in the stress phase only or in both phases, stress and rest phases. SPECT: single-photon emission computed tomography; ^18^F-FDG: fluorodeoxyglucose F18; PET: positron-emission tomography

### Prognosis:

Observational studies have suggested that the presence of viable myocardium is directly associated with favorable progress of left ventricular function and good prognosis after revascularization. Patients who seem to benefit more from surgical revascularization are those with ischemic symptoms and severe left ventricular dysfunction. A significant perioperative risk should be considered in relation to long-term benefits on mortality.^62-66^

### Comparison of randomized studies of miocardial viability

Today, there is little evidence of randomized studies on this theme, with conflicting results.

### Stich trial

Randomized, multicenter study involving 1,212 patients, 601 assessed for myocardial viability by dobutamine echocardiography (130 patients), SPECT (321 patients) or both (150 patients).^67^ In the myocardial viability study, 298 patients were randomly allocated to receive conservative treatment plus surgical revascularization, and 303 patients to receive pharmacological therapy alone. Median follow-up period was 56 months (12 months - 100 months).^67^ No statistically significant benefit of surgical intervention on mortality, or of assessment of myocardial viability on surgical intervention, suggesting that investigation of a viable myocardium do not differentiate patients who would benefit from revascularization from those who would benefit from medical therapy alone.^67^

Despite its limitations and biases, the STICH trial is, so far, the largest study on the influence of myocardial viability on clinical outcomes in patients with ischemic heart disease. Also, it is the first study to evaluate differential results of revascularization and pharmacological therapy.^67^

## PARR-2 Trial

Study designed to evaluate the efficacy of ^18^F-FDG PET in patients with left ventricular dysfunction, by risk stratification and identification of those who would benefit from myocardial revascularization. A total of 430 patients with LVEF < 35% and CAD were allocated into two groups - standard care (n = 212) and treatment assisted by ^18^F-FDG PET (n = 218).^68^

At one year, the PARR-2 trial did not show a significant difference between the groups in the primary outcomes that included death for cardiac causes, acute myocardial infarction or hospital stays for cardiac cause (30% vs. 36% p = 0.15). In PET group, however, there was a significant decrease in primary outcome over the follow-up period (relative risk 0.62; 95% CI 0.42 - 0.93; p = 0.019).^68^

### Perspectives

Myocardial viability is still a subject of clinical importance and a focus of clinical trials and translational science. Pathophysiological basis of left ventricular ischemic dysfunction seems to be correlated with myocardial stunning, hibernation or myocardial necrosis. Imaging methods used for assessment of viable muscular tissue have their own operational characteristics and should be appropriate to the patient’s individual characteristics. The detection of myocardial viability may be a valuable predictor of the response to revascularization and long-term prognostic and, thereby, contribute to the decision-making in the medical practice.

^18^F-FDG PET and CMR are considered first-choice methods for detection of viability due to their high sensitivity and specificity rates, whereas both echocardiography and myocardial scintigraphy considered acceptable methods for their wide availability and accessibility. With respect to the impact on medical practice, there are no definite studies showing the benefits of myocardial viability assessment on patients’ prognosis, which reinforce the necessity of larger studies, considering the great relevance of the theme.
